# Toward Elimination of Soot Emissions from Jet Fuel Combustion

**DOI:** 10.1021/acs.est.3c01048

**Published:** 2023-07-05

**Authors:** Georgios A. Kelesidis, Amogh Nagarkar, Una Trivanovic, Sotiris E. Pratsinis

**Affiliations:** † Particle Technology Laboratory, Institute of Energy and Process Engineering, Department of Mechanical and Process Engineering, ETH Zürich, Sonneggstrasse 3, CH-8092 Zürich, Switzerland; ‡ Nanoscience and Advanced Material Center, Environmental and Occupation Health Science Institute, School of Public Health, Rutgers University, Frelinghuysen 170 Piscataway, New Jersey 08854, United States

**Keywords:** jet fuel combustion, soot, oxidation, morphology, nanostructure

## Abstract

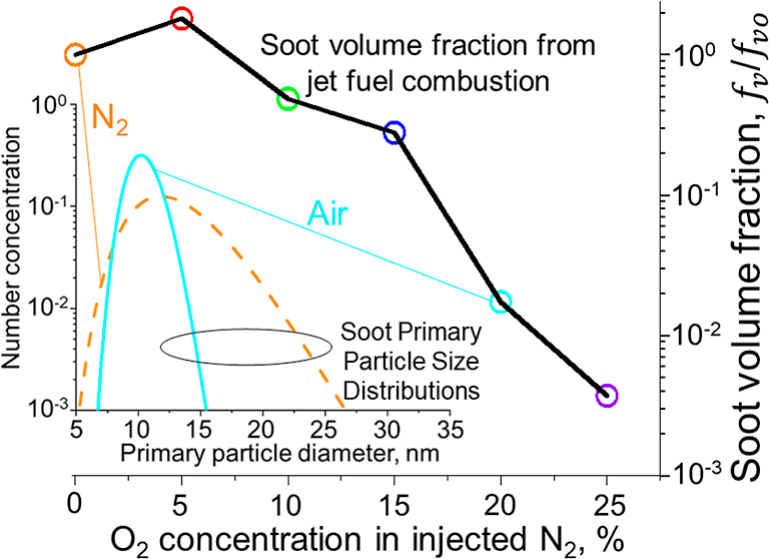

Soot from jet fuel combustion in aircraft engines contributes to global warming through the formation of contrail cirrus clouds that make up to 56% of the total radiative forcing from aviation. Here, the elimination of such emissions is explored through N_2_ injection (containing 0–25 vol % O_2_) at the exhaust of enclosed spray combustion of jet fuel that nicely emulates aircraft soot emissions. It is shown that injecting N_2_ containing 5 vol % of O_2_ enhances the formation of polyaromatic hydrocarbons (PAHs) that adsorb on the surface of soot. This increases soot number density and volume fraction by 25 and 80%, respectively. However, further increasing the O_2_ concentration to 20 or 25 vol % enhances oxidation and nearly eliminates soot emissions from jet fuel spray combustion, reducing the soot number density and volume fraction by 87.3 or 95.4 and 98.3 or 99.6%, respectively. So, a judicious injection of air just after the aircraft engine exhaust can drastically reduce soot emissions and halve the radiative forcing due to aviation, as shown by soot mobility, X-ray diffraction, Raman spectroscopy, nitrogen adsorption, microscopy, and thermogravimetric analysis (for the organic to total carbon ratio) measurements.

## Abstract 2

Injection of air at the exhaust of enclosed spray combustion of jet fuel nearly eliminates soot emissions and can be used to guide halving the contribution of aviation to global warming.

## 1 Introduction

About a million tons of carbonaceous (soot) nanoparticles are released every year by aviation through incomplete combustion of jet fuel.^1^ These emissions have a major impact on the health of airport workers and communities living near airports due to their cytotoxicity.^2^ In addition, soot nanoparticles typically form clusters (agglomerates) that strongly absorb light, reducing visibility and increasing the radiative forcing, RF, and thus the Earth’s temperature.^3^ Most importantly, aircraft soot emissions act as ice nuclei and form contrail cirrus clouds.^4^ The RF from such contrails makes up about 56% of the total RF induced by aviation.^5^ Thus, eliminating soot emissions from aircraft engines is essential to limit their impact on public health and substantially reduce their climate forcing.^6^

To this end, bio-based (e.g., hydrotreated esters and fatty acids, HEFA^7^) or synthetic fuels derived by the Fischer–Tropsch (FT) process^8^ have been explored to reduce the soot emissions from the combustion of petroleum-based jet fuels in aircraft engines. For example, a 50:50 blend of jet A and HEFA fuels decreased the total number concentration, *N*_t_, of soot nanoparticles^7^ by 50–70%. Similarly, the combustion of a 60:40 blend of jet A1 and FT-derived fuels lowered by 34–50% the soot *N*_t_.^8^ Blending jet fuel with such alternative fuels decreases the mean mobility,^7^, and primary particle diameters,^9^, of soot by about 15 and 30%, respectively. Raman and microscopy analyses indicate that the combustion of biofuels results in more amorphous soot than jet fuels, while FT-derived fuels yield more graphitic soot.^9^

Despite the rather large (50–70%) reduction of aircraft soot emissions, using blends of jet with bio-based or synthetic fuels reduces only up to 20% the RF from contrail cirrus clouds.^6^ In this regard, climate modeling revealed that a 90% decrease of soot *N*_t_ can reduce this RF^6^ up to 50%. This can be attained through gas (or air) injection downstream of the aircraft combustors.^10^ For example, the design of quite a few of the current aircraft combustors is based on the rich quench lean (RQL) concept^11^ where swirling and cross-flow jets are used in the primary zone to produce high concentrations of soot.^12^ This zone is followed by a lean dilution zone, where additional air is injected to oxidize that soot.^12^ Similarly, O_2_ was introduced downstream of model laboratory RQL combustors burning ethylene^13^ to oxidize soot and reduce its volume fraction,^14^*f*_v_, and *N*_t_ up to 99%. However, soot produced by ethylene combustion contains a higher organic and amorphous carbon content than aircraft soot from jet fuel combustion.^15^ In particular, Raman spectroscopy showed that the oxidative reactivity of soot increases with its amorphous carbon content.^16^ Recently, the impact of air injection downstream of jet fuel combustion was elucidated in a laboratory RQL combustor.^17^ The rather small air flow rates used there resulted in low O_2_ concentrations downstream of the combustor^17^ that reduced soot *f*_v_ up to 40%. The limited reduction of soot *f*_v_ in current RQL combustors can be attributed to the inhomogeneity of temperature and gas profiles that result in regions with high concentrations of soot that survive oxidation and exit the combustor.^18^ Large reductions (>90%) of soot *N*_t_ and *f*_v_ have been attained by dilution and combustion of ethylene^19^ or jet fuel^20^ soot in a lean premixed flame.^21^ However, this exhaust treatment system does not resemble the dilution zones in common RQL combustors.^18^ Similarly, “soot-free” combustion of jet fuel was attained recently in a laboratory-scale lean azimuthal flame (LEAF) combustor of jet A1 fuel by enhancing soot oxidation while injecting hydrogen.^22^

Here, enclosed spray combustion (ESC) of jet fuel (Figure S1) that produces surrogate aircraft soot emissions^23^ is used to explore their elimination. During ESC of jet fuel, soot nanoparticles grow by surface reactions^24^ and agglomeration,^25^ attaining similar morphology, size distribution, and organic carbon content with those of aviation emissions.^23^ Most importantly, the Raman spectrum of soot from ESC of jet fuel is in excellent agreement with that measured from aircraft soot^26^ (Figure S2). This indicates that the oxidative reactivity of such surrogate aircraft soot is similar to that of aviation emissions.^16^ So, the elimination of such soot is investigated here by injecting N_2_ containing 0–25 vol % of O_2_ downstream of ESC of jet fuel. The impact of such O_2_ addition on the soot mobility, primary particle size distributions, *f*_v_, *N*_t_, composition, and nanostructure is elucidated below for the first time to the best of our knowledge. That way, the transformation of soot during oxidation is quantified, providing a basis for optimization of the RQL concept that is already used by some aircraft engine manufacturers.^11^

## 2 Materials and Methods

Soot nanoparticles were generated by ESC. Briefly, soot was produced by jet A fuel (POSF 10325^27^) spray combustion using an external-mixing, twin fluid nozzle^28^ enclosed in two, 30 cm long quartz tubes (each with a 42 mm inner diameter) in series^29^ (Figure S1). So, 4 mL/min of fuel was dispersed into a fine spray with 1 L/min of O_2_. The resulting spray was ignited and sustained by a supporting premixed methane/oxygen flame (CH_4_ = 1.25 L/min, O_2_ = 2.25 L/min). Sheath air was fed through 12 evenly spaced holes surrounding the spray flame at 17.2 L/min. A torus ring^30^ with 12 jet outlets between the two tubes (height above burner, HAB = 30 cm) was used to introduce 20 L/min of N_2_ with or without O_2_ in an upward swirled pattern to quench the flame as well as to dilute and oxidize the exhaust soot emissions. The O_2_ concentration, [O_2_], was varied from 0 to 25 vol %. The steel torus ring was made using two pieces of pipe welded to a tube (Figure S3) having a 0.38 cm inner diameter and 12 outlets, each having a 0.06 cm diameter^30^ with an upward azimuth angle of 10°.

**Figure 1:**
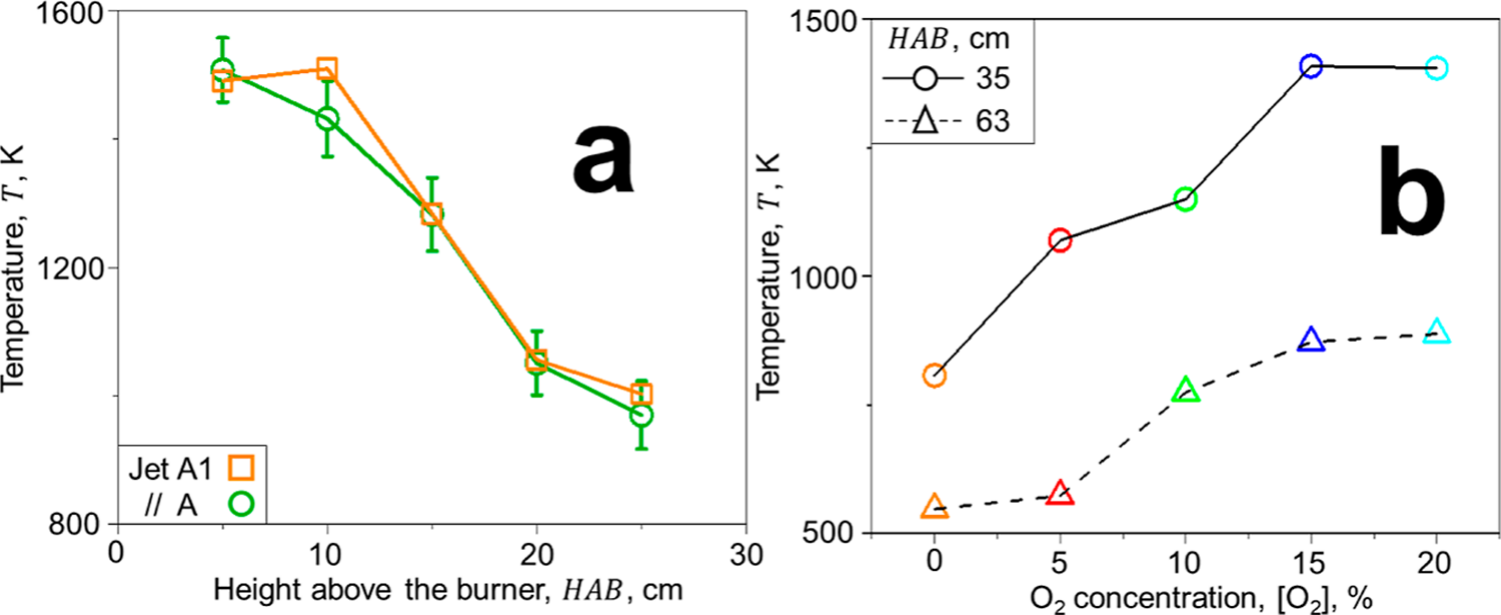
Centerline temperature (a) by ESC of jet fuel A (circles) and A1 (squares) as a function of HAB, and (b) by ESC of jet fuel A1 as a function of [O_2_] in the injected N_2_ jets from the torus ring at HAB = 35 (circles) and 63 cm (triangles).

The temperature profile, *T*, was measured using a 1 mm (nominal) bead diameter and an R-type thermocouple (Intertechno-Firag AG) and corrected for radiative heat losses.^31^ The *T* measurements and energy balance used here have been described and validated for ESC of jet A1 fuel.^31^ The centerline flame *T* profiles during ESC of jet A (Figure 1a: circles) and A1 (squares) fuel are quite similar. Figure 1b shows that the centerline *T* by ESC of jet A1 fuel at HAB = 35 (circles) and 63 cm (triangles) increases with increasing oxygen content in the injected nitrogen jets from the torus ring at HAB = 30 cm, as expected.

Soot was extracted from the centerline of the flame at HAB = 63 cm using a straight tube sampler.^32^ The sampled aerosol was rapidly diluted and quenched by mixing with N_2,_ followed by compressed air from a rotating disk diluter. The total dilution factor was set to 33.24 at all conditions investigated here. The distribution of the soot mobility diameter, *d*_m_, and its total number concentration, *N*_t_, were obtained by averaging five 65 s scans of a scanning mobility particle sizer.^32^ The soot *f*_v_ was estimated based on the measured *d*_m_ and *d*_p_ distributions, accounting for the soot agglomerate structure:^33^
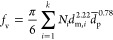
1where *N*_*i*_ is the number concentration of soot agglomerates having *d*_m,*i*_ and mean . The index *k* varies from 1 to 100, i.e., the largest number of *d*_m_ bins measured by the scanning mobility particle sizer. The exponents for  and *d*_m,*i*_ in eq 1 were validated with aerosol particle mass analyzer data in premixed,^34^ diffusion^25^ and spray^23^ flames. Equation 1 has been derived by capitalizing on a power law for the soot effective density that was obtained by discrete element modeling of soot agglomeration and surface growth.^25^ This equation has been used to measure accurately the soot *f*_v_ in laminar premixed,^35^ diffusion flames, and diesel engines,^36^ accounting for the realistic morphology of soot.^33^

Soot was also collected on a glass fiber filter for off-line analysis. Then, Raman spectra of such soot nanoparticles were obtained using a 515 nm laser having 50 mW power (Renshaw inVia). The laser was focused with a ×20 magnification optical microscope, which gives a 2 μm spot size, while a 10% laser power was focused on the sample for 120 s and three acquisitions.^37^ The intensities of the disorder (*D* ∼ 1350 cm^–1^) and graphitic (*G* ∼ 1580 cm^–1^) bands^37^ were obtained after straight line subtraction of the baseline.^38^

The X-ray diffraction (XRD) patterns of soot at diffraction angles, 2θ = 10–70°, were also obtained by an AXS D8 diffractometer (Bruker) at a scan rate of 0.0197°/s. Here, the average interlayer distance, *d*, of soot was obtained by analyzing the 002 XRD peak using Bragg’s law^39^
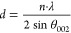
2where *n* = 1 is the order of diffraction, λ = 0.154 nm is the wavelength of the diffractometer, and θ_002_ is the center angle of the 002 peak. Similarly, the average crystallite length, *L*_c_, of spray flame soot was obtained by^39^
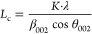
3where *K* = 0.89 is the peak shape factor^40^ and β_002_ is the full width of the half maximum of the 002 peak. The crystallites *d* and *L*_c_ were determined here using eqs 2 and 3 with the θ_002_ and β_002_ derived from the measured XRD patterns that were validated using the patterns of commercial carbon blacks.^41^

The organic to total carbon (OC/TC) mass ratio of soot was obtained by thermogravimetric analysis (TGA).^42^ The samples were first placed in N_2_ to volatilize OC and then in air to oxidize the elemental carbon (EC). The sample heating began at 30 °C in N_2_ and was ramped up to 900 °C at 20 °C/min. The temperature was held at 900 °C for 10 min before dropping back to 30 °C at 20 °C/min. The same temperature profile was then repeated in air. From the TGA mass loss, the OC/TC was estimated as the ratio of mass lost under N_2_ divided by the total mass lost in both stages.

Soot nanoparticles were analyzed by N_2_ adsorption on a Tristar II Plus surface area and a porosity system (Micromeritics) at 77.3 K after degassing in vacuum (VacPrep 061, Micromeritics) at 200 °C overnight. The specific surface area, SSA, was derived from N_2_ adsorbed at five relative pressures ranging from 0.05 to 0.25 using the Brunauer–Emmett–Teller method.^43^

Soot nanoparticles were also imaged using transmission electron microscopy (TEM, FEI Tecnai F30 FEG). The nanoparticles were dispersed in ethanol and placed in an ultrasonic bath for 15 min to break up large agglomerates.^23^ A drop of ethanol solution was then placed on lacey carbon TEM grids with a 200 mesh copper support (LC200-Cu-150, Electron Microscopy Sciences) and allowed to dry. The primary particle diameter, *d*_p_, was measured by manually placing ellipses over the primary particles in ImageJ^44^ and calculating the area-equivalent diameter. About 150–200 primary particles were counted for each [O_2_] condition to obtain statistically significant size distributions.^23^

## 3 Results and Discussion

### 3.1 Reducing Surrogate Aviation Soot Emissions by O_2_-Containing Jets

Extensive recirculation results in radially rather uniform conditions away from the burner, as has been shown for temperature, *T*, by computational fluid dynamics (CFD) analysis (e.g., Figure 1a in ref (45)). To further confirm this for the soot aerosol, its average mobility diameter, , *f*_v_, and *N*_t_ were measured at the centerline (*r*/*R* = 0) and in-between the tube wall and centerline (*r*/*R* = 0.5; Table S1) at HAB = 25 cm (i.e., well below the location of the torus ring with the 12 N_2_-jets containing O_2_). The soot *N*_t_, *f*_v_, and  at the centerline are similar (within the measurement variation) to those obtained in-between the tube wall and centerline there. This indicates that the soot aerosol has been largely homogenized across the tube radius when it reaches the torus ring (HAB = 30 cm). Further downstream, the soot size distribution becomes even more uniform across the tube due to its intense mixing with the O_2_-containing N_2_ jets, as shown in Figure S4 for two radial locations at HAB = 35 and 63 cm, as well as by the corresponding *N*_t_ and mean  (Table S2). This indicates that the soot aerosol is well mixed across the enclosing tube, corroborating CFD simulations at similar gas-aerosol mixing configurations.^30^

**Figure 2:**
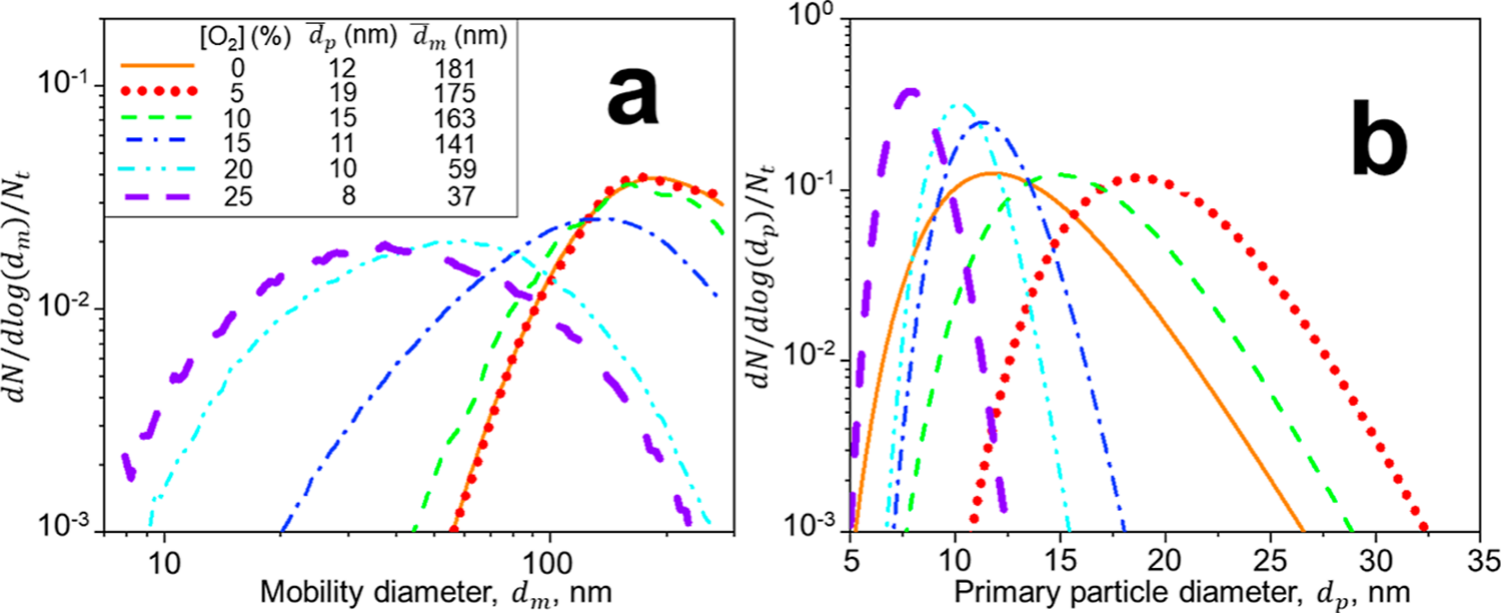
Impact of O_2_-containing N_2_ jets on soot characteristics. Mobility (a) and primary particle (b) size distributions along with the mean mobility, , and primary particle, , diameters of soot from ESC of jet fuel and mixed with N_2_ jets having O_2_ concentrations, [O_2_] = 0 (solid line), 5 (dotted line), 10 (broken line), 15 (dot-broken line), 20 (double dot-broken line), and 25 vol % (thick broken line).

Figure 2 shows the soot mobility (a) and primary particle (b) size distributions at the centerline of HAB = 63 cm along with their mean soot  and  from ESC of jet fuel and mixed with N_2_ jets containing 0–25 vol % O_2_. In the absence of oxidation, ([O_2_] = 0 vol %), soot nanoparticles form large agglomerates that have a broad *d*_m_ distribution with mean  = 181 nm (Figure 2a: solid red line), in good agreement with those measured from ESC of jet A1 fuel at similar equivalence ratios.^23^ The primary particles making up these agglomerates have a relatively narrow size distribution with a geometric standard deviation, σ_g_ = 1.27 with  = 12 nm (Figure 2b: solid red line). Increasing [O_2_] to 5 vol % hardly alters the soot mobility size distribution (dotted line). In contrast, the primary particle size distribution shifts to larger *d*_p_, consistent with the literature on low O_2_ (<10 vol %) addition that enhances the formation of PAHs^46^ through the generation of reactive O_2_ species.^47^ Most likely, the increase of soot  at [O_2_] = 5 vol % can be attributed to such PAHs that adsorb on the soot surface (as confirmed here by TGA and Raman spectroscopy, Figure 4f,h). The mobility and primary particle size distributions measured here for soot from ESC of jet fuel at [O_2_] = 5 vol % are consistent with those measured for soot made in laminar flow reactors at low O_2_ concentrations.^47^ Increasing the O_2_ concentration in the injected N_2_ jets increases the flame *T* at HAB = 35 cm from 780 K at [O_2_] = 0 vol % up to 1400 K at [O_2_] = 20 vol % (Figure 1b). At such a high *T*, surface oxidation takes place^48^ reducing both soot *d*_m_ and *d*_p_. In particular, increasing [O_2_] up to 20 and 25 vol % enhances soot oxidation, reducing its  to 59 and 37 nm and its  to 10 and 8 nm. The broad *d*_m_ distributions at large [O_2_] are similar to those obtained after diluting and combusting ethylene^19^ and jet fuel^20^ soot with air in lean premixed flames. These broad distributions can be attributed to fragmentation by oxidation suggested by measurements and simulations of diesel soot oxidation.^49^ Furthermore, the *d*_p_ distribution narrows drastically by surface oxidation at large [O_2_], i.e., from 1.27 at [O_2_] = 0 vol % down to σ_g_ of 1.13 and 1.14 at [O_2_] = 20 and 25 vol %, respectively.

**Figure 3:**
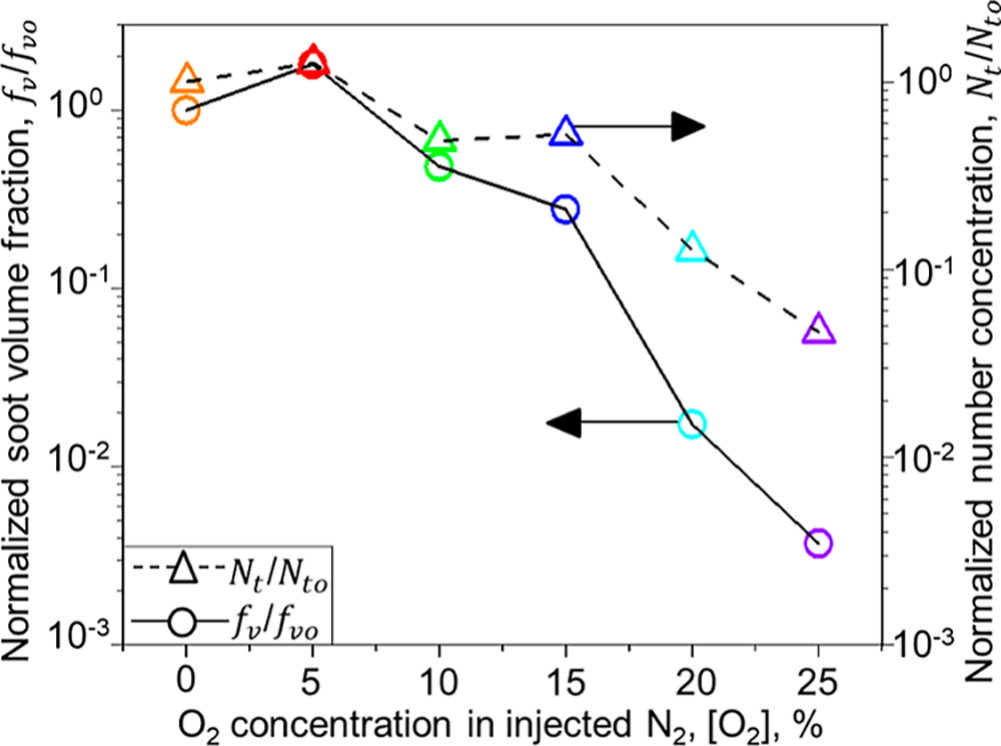
Reducing soot emissions by downstream injection of O_2_-containing N_2_. Normalized volume fraction, *f*_v_/*f*_vo_ (circles and a solid line), and total number density, *N*_t_/*N*_to_ (triangles and a broken line), of soot produced from ESC of jet A fuel and mixed downstream with 20 L/min of O_2_-containing N_2_ jets as a function of their [O_2_] normalized by the *f*_vo_ = 1.4 × 10^–8^ and *N*_to_ = 6.7 × 10^7^ cm^–3^ at [O_2_] = 0 vol %.

The mobility and primary particle size distributions measured here can be used to obtain the *N*_t_ (Figure 3: triangles and a broken line) and *f*_v_ (circles and a solid line). The latter is derived by accounting for the realistic agglomerate structure of soot that is essential to close its mass balance.^33^ Increasing [O_2_] from 0 to 5 vol % enhances soot *f*_v_ by 80% (Figure 3) due to the PAH formation and adsorption on the soot surface,^46^ consistent with the soot *f*_v_ increase after injection of small amounts of air downstream of synthetic fuel combustion.^17^ Soot *N*_t_ also increases by 25%. This could be attributed to the inception of nascent soot enabled by the low concentrations^47^ of O_2_. Increasing [O_2_] to 20 vol % almost eliminates soot emissions by reducing *f*_v_ and *N*_t_ by 98.3 and 87.3%, respectively. Further increasing [O_2_] to 25 vol % hardly affects *f*_v_ and *N*_t_, reducing them by 99.6 and 95.4%, respectively. The reduction of soot *N*_t_ obtained here is on par with the 99.9% *N*_t_ reduction measured after air dilution and combustion in a lean premixed flame.^20^ This indicates that rather uniform soot concentration profiles are attained here (Figure S4 and Table S2), similar to those in premixed flames.^20^

Furthermore, the soot *f*_v_ reduction measured here is 50% larger than that attained in a laboratory-scale RQL combustor.^17^ This could be attributed to potentially more homogeneous mixing of soot with oxidizing gas by employing the current jet configuration. The soot *f*_v_ = 5 × 10^–11^ obtained here at [O_2_] = 25 vol % is on par with the *f*_v_ = 3 × 10^–11^ to 6 × 10^–11^ measured in a so-called “soot-free” LEAF combustor.^22^ In fact, the corresponding *N*_t_ = 3.1 × 10^6^ #/cm^3^ is 3 orders of magnitude lower than the *N*_t_ = 3.5–7.5 × 10^9^ #/cm^3^ measured in LEAF.^22^ The largest 95.4% *N*_t_ reduction of jet fuel emissions attained here using O_2_-containing N_2_ jets is about 25–60% greater than that obtained by blending jets with HEFA^7^ or FT-derived^8^ fuels.

### 3.2 Soot Nanostructure and Composition

**Figure 4:**
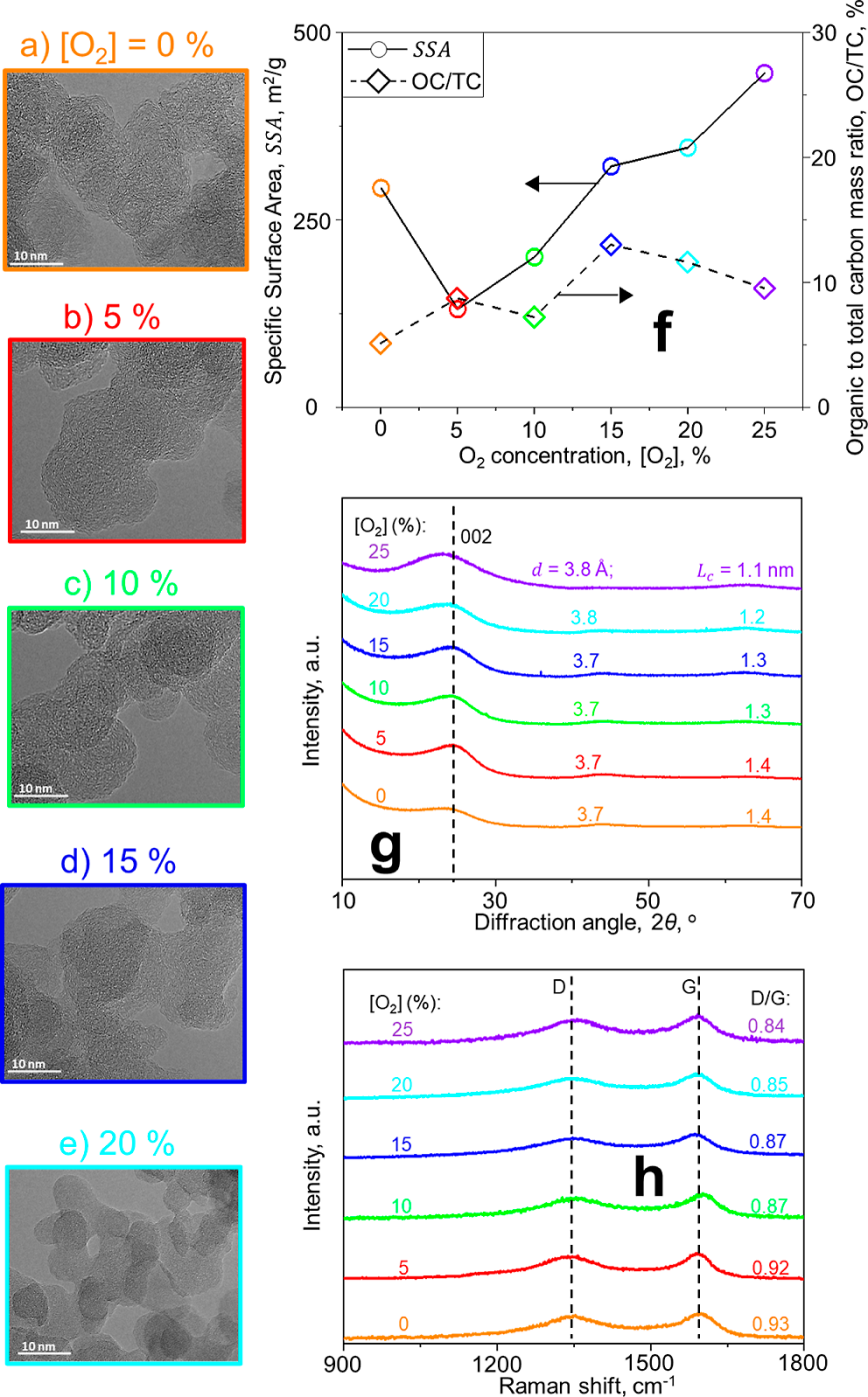
Characterization of soot nanostructure and composition. Microscopy images (a–e), specific surface area, SSA (f: left ordinate), and organic to total carbon (OC/TC) mass ratio (f: right ordinate); XRD patterns (g) and Raman spectra (h) of soot from ESC of jet fuel and mixed with N_2_ jets having [O_2_] of 0–25 vol %.

Even though the large reduction of soot *N*_t_ and *f*_v_ using O_2_-containing dilution jets is promising, the nanostructure and composition of the remaining soot emissions have to be characterized to assess their impact on public health and climate. In this regard, Figure 4a–f show microscopy images of soot produced here by ESC of jet fuel and diluted with O_2_-containing jets having [O_2_] = 0 (a), 5 (b), 10 (c), 15 (d), and 20 vol % (e). In the absence of additional O_2_ at the exhaust ([O_2_] = 0 vol %), small and rather graphitic soot nanoparticles are formed (a). At [O_2_] = 5 vol % (b), probably polyaromatic hydrocarbons (PAHs) are generated^46^ that adsorb onto the soot surface and increase the primary particle diameter. Further increasing [O_2_] up to 20 vol % (e) enhances the oxidation of soot nanoparticles, reducing their diameter and making them more amorphous.

Figure 4f shows the specific surface area, SSA (circles and a solid line), and organic to total carbon (OC/TC) mass ratio (diamonds and a broken line) of soot produced here at the conditions shown in Figure 3. The SSA of soot correlates with its cytotoxicity^50^ and thus it is essential to quantify its impact on public health. At [O_2_] = 0 vol %, soot nanoparticles have SSA = 292.3 m^2^/g and OC/TC = 5.1%, consistent with those measured from ESC of jet A1 fuel at similar equivalence ratios.^23^ Varying [O_2_] from 0 to 5 vol % decreases the SSA of soot to 130.6 m^2^/g and increases its OC/TC to 8.7%, as small amounts of O_2_ enhance PAH formation^46^ and thus the soot OC/TC. As [O_2_] further increases up to 25 vol %, soot nanoparticles are oxidized and their diameter decreases (as discussed in Figure 2), increasing their SSA up to 445.5 m^2^/g. This 50% enhancement of soot SSA attained here is on par with the 30% increase obtained by blending jet with alternative fuels.^9^ Introducing dilution jets with [O_2_] more than 5 vol % enhances slightly the adsorption of PAHs and increases the OC/TC of the soot up to 10–13%. This can reduce the light absorption of soot^51^ and thus its direct radiative forcing^3^ by up to 17%. Atmospheric transformations of particle composition and morphology (e.g., during water processing^52^) should be accounted for to most accurately quantify the impact of aircraft soot emissions on public health and climate.

The impact of O_2_-containing N_2_ dilution jets on soot nanostructure is quantified by X-ray diffraction (XRD) and Raman spectroscopy. Figure 4g shows the XRD patterns along with the mean interlayer distance, *d*, and crystallite length, *L*_c_, of soot produced at various [O_2_]. The pattern of unoxidized ESC soot ([O_2_] = 0 vol %) exhibits a rather broad 002 peak (broken line) at a diffraction angle, 2θ, of about 24° that yields *d* = 3.7 Å and *L*_c_ = 1.4 nm, in agreement with the XRD pattern of unoxidized carbon black^41^ and aircraft soot.^26^ Surface oxidation at [O_2_] = 5–15 vol % hardly affects *d* and *L*_c_ of soot, consistent with the XRD patterns of carbon black oxidized at similar O_2_ concentrations.^41^ Further increasing [O_2_] to 20–25 vol % shifts the peak to smaller diffraction angles, increasing *d* to 3.8 Å and reducing *L*_c_ to 1.2–1.1 nm. This indicates that oxidation at such large [O_2_] makes soot less graphitic, more amorphous, and subsequently more reactive.^16^

Figure 4h shows the Raman spectra along with the mean ratio of the disorder (D) over the graphitic (G) band of soot produced at various [O_2_]. Increasing [O_2_] from 0 to 5 vol % hardly alters the nanostructure and the Raman spectrum of soot. However, further increasing [O_2_] from 5 to 20 and 25 vol % reduces D/G to 0.85 and 0.84. This D/G reduction indicates that the average PAH size of soot decrease^53^ due to oxidation and small PAH adsorption, consistent with Raman spectroscopy measurements of oxidized carbon black,^54^ and soot from premixed^55^ and diffusion^56^ flames. The small PAH sizes attained after soot oxidation with [O_2_] = 20 vol % enhance the oxidative reactivity of soot^16^ and thus its reactions with ozone in the atmosphere.^57^ Most importantly, the amorphous soot emitted after such oxidation has smaller ice nucleation activity than graphitic soot^58^ produced in the absence of downstream O_2_ here. This can further limit the formation of contrail cirrus clouds and thus their radiative forcing!

### 3.3 Discussion and Outlook

In conclusion, it is shown that injecting air downstream of jet fuel combustion can drastically reduce its soot emissions. By capitalizing on the quantitative understanding of soot oxidation^48^ and, in particular, surface growth and agglomeration dynamics in the ESC reactor and torus ring,^31^ it was shown that upward injection of 12 swirling O_2_-containing N_2_ jets facilitates close contact of the soot aerosol with oxidizing gas to enable drastic reduction of soot emissions (Figure 3). In particular, the injection of N_2_ containing 20–25 vol % of O_2_ enhances the oxidation of soot nanoparticles and decreases their *N*_t_ and *f*_v_ by 87.3–95.4 and 98.3–99.6%, respectively. Oxidation at these conditions increases the amorphous and organic carbon content of the emitted soot, reducing its light absorption,^51^ direct radiative forcing,^3^ and ice nucleation activity.^58^ The number concentration of ice nuclei formed in the contrails of aircraft engines decreases almost linearly as the soot number concentration decreases from 10^16^ down to about 8 × 10^13^ #/kg of fuel.^4^ Recent measurements have shown that aircraft engines combusting jet A1 fuel release 5 × 10^15^ #/kg of fuel (see Figure 4 in ref (8)). Injection of O_2_ downstream of jet A or A1 fuel combustion reduces the soot *N*_t_ up to about an order of magnitude (Figure 3). In this *N*_t_ range, the concentration of ice nuclei seems to decrease linearly with the soot concentration.^4^ This suggests that the injection of air downstream of aircraft engines may reduce the radiative forcing from their emissions^6^ by at least 50%.

To relate the present results to the emissions of actual jet engines, besides matching fuel and oxidant composition (jet fuel A or A1 and air or [O_2_] = 20%), one has to match the so-called high temperature particle residence time between ESC and jet engines, as has been shown in the combustion synthesis of nanoparticles (i.e., Figure 7 in ref (59)). The scale-up of the present spray combustion reactor has been explored experimentally and numerically^59^ up to 2 orders of magnitude^60^ where it was shown that the characteristics of flame-made nanoparticles can be preserved by maintaining similar high-temperature particle residence times across scales. In this regard, the present set of data is essential to derive and validate CFD^59^ and moving sectional models for soot oxidation from jet fuel combustion.^48^ Such models can be used to obtain robust oxidation rates for aircraft soot emissions and facilitate the design and scale-up of engine exhausts with minimal, if not zero, soot emissions.

## Supporting Information Available

Schematic of the experimental setup for the generation and elimination of soot from ESC of jet fuel; Raman spectra of soot from ESC of jet fuel and aircraft engines operating at two thrusts;^25^ top and side views of the torus ring for injection of O_2_-containing N_2_ along with a reference ruler; mobility size distributions of soot from ESC of jet A1 fuel and controlled oxidation at the flame centerline (*r*/*R* = 0, dotted lines) and in-between the enclosing tube wall and centerline (1 cm away from each, *r*/*R* = 0.5; solid lines) at HAB = 35 and 63 cm along with the corresponding total particle concentration, *N*_t_; and soot *N*_t_, *f*_v_, and  at the centerline and in-between there and tube wall 5 cm below and above the torus ring as well as at HAB = 63 cm (PDF)
