# Supplementary material for: Toward Elimination of Soot Emissions from Jet Fuel Combustion
Source: Environ Sci Technol. 2023 Jul 5;57(28):10276–83. doi: 10.1021/acs.est.3c01048 (PMC10357563; doi:10.1021/acs.est.3c01048)
Supplement: Supplementary file 1 — es3c01048_si_001.pdf [file es3c01048_si_001.pdf]

Supplementary Information for

Towards elimination of soot emissions from jet fuel combustion

Georgios A. Kelesidis[1,2], Amogh Nagarkar[1], Una Trivanovic[1] and Sotiris E. Pratsinis[1,]*

[1]Particle Technology Laboratory, Institute of Energy and Process Engineering, Department of Mechanical and Process Engineering, ETH Zürich, Sonneggstrasse 3, CH-8092 Zürich, Switzerland.

[2]Nanoscience and Advanced Material Center, Environmental and Occupation Health Science Institute, School of Public Health, Rutgers University, Frelinghuysen 170, 08854 Piscataway, NJ, USA.

*Corresponding author: sotiris.pratsinis@ptl.mavt.ethz.ch

5 Pages,

2 Tables

& 4 Figures

Summary:

Figure S1………………………………………………………………………...p. S2

// S2………………………………………………………………………...p. S3

// S3……………………………………………………………………..….p. S3

// S4…………………………………………………………..……….p. S4

Table S1……………………………………………………………...……p. S4

// S2…………………………………………………………...……….p. S4

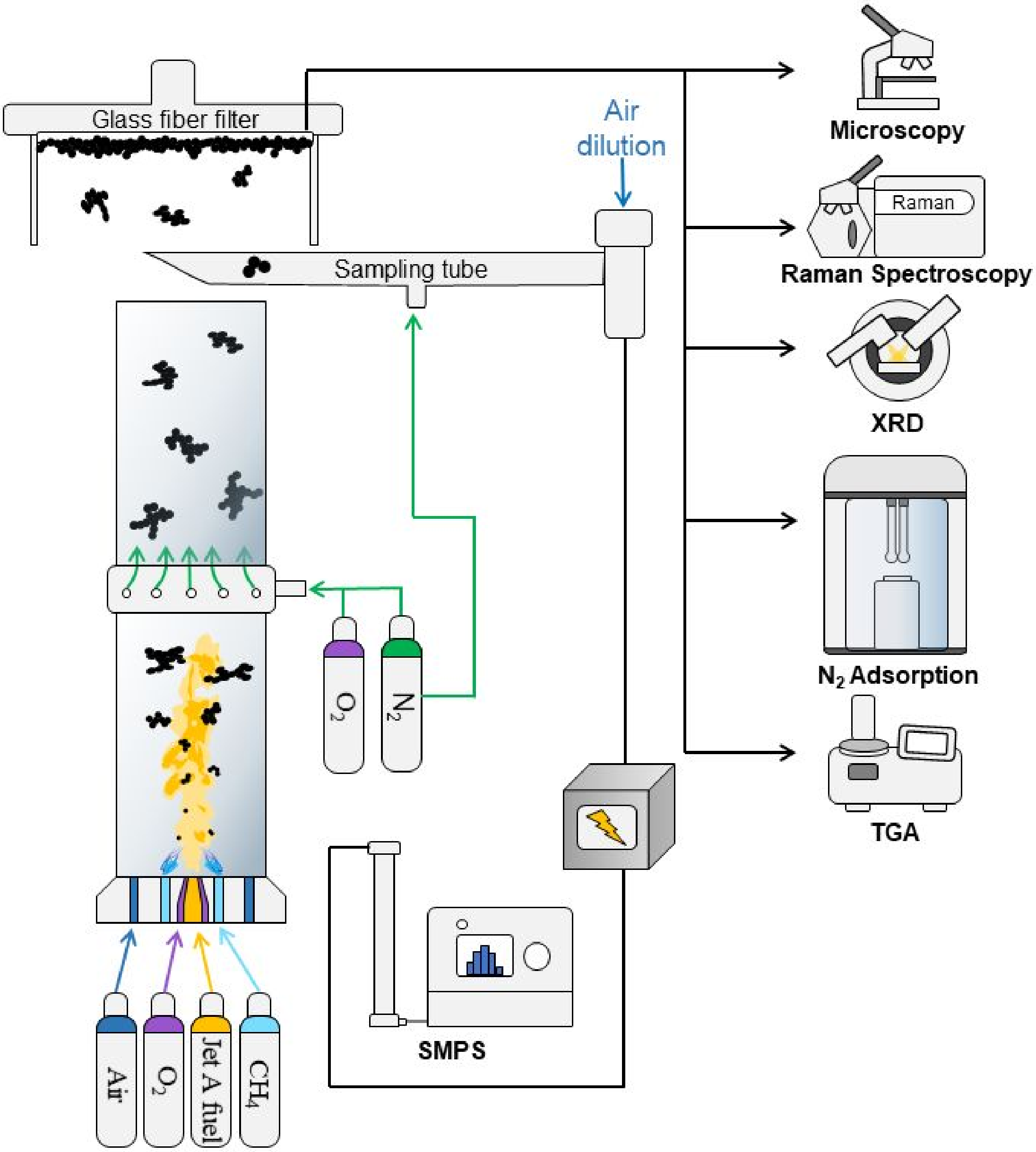

Figure S1. Schematic of the experimental set up for generation and elimination of soot from enclosed spray combustion (ESC) of jet fuel. Jet A fuel is atomized and combusted using an external twin fluid nozzle[28] enclosed in two, 30 cm long quartz tubes in series[29]. A torus ring[30] with 12 jet outlets between the two tubes was used to introduce 20 L/min of N_2 with $[O_2]$ = 0 - 25 vol %. Particles are sampled at various heights above the burner (HAB) using a straight tube sampler and diluted with nitrogen and air using a rotating dilution system[32]. The diluted soot aerosol flows through an X-ray neutralizer followed by a scanning mobility particle sizer (SMPS). Soot nanoparticles are also deposited on a glass fiber filter at the top of the unit. The collected soot is analyzed offline by microcopy, N_2 adsorption, Raman spectroscopy, X-ray diffraction (XRD) and thermogravimetric analysis (TGA)[23].

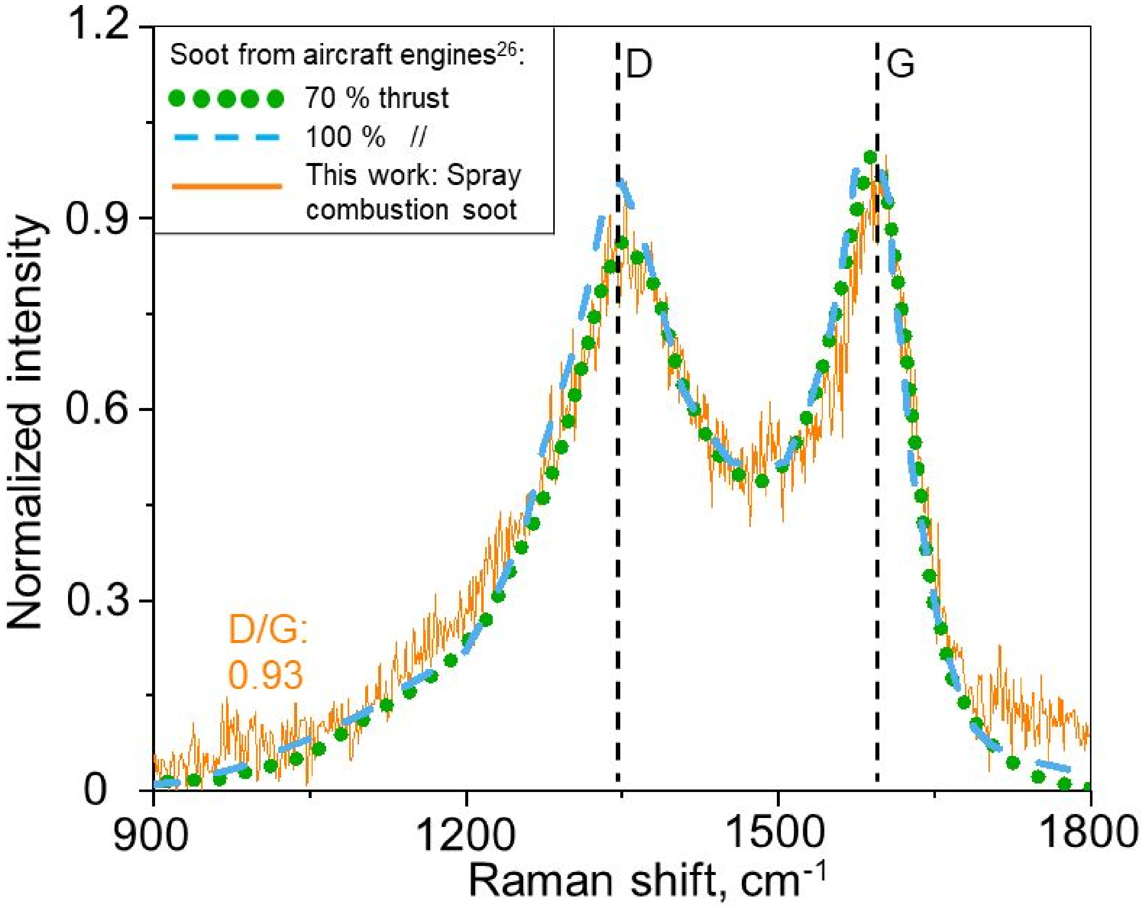

Figure S2. Raman spectra of soot from ESC of jet fuel and aircraft engines operating at two thrusts[26]. The Raman spectrum of ESC soot exhibits disorder (D) and graphitic (G) bands (broken lines) having ratio D/G = 0.93. This D/G is nearly the same with the D/G = 0.95 ± 0.05 for mature soot from premixed ethylene flames[S1]. Most importantly, the Raman spectrum of soot from ESC here is in excellent agreement with that measured from aircraft soot[26] indicating that the oxidative reactivity of such surrogate aircraft soot[23] is similar to that of aviation emissions[16].

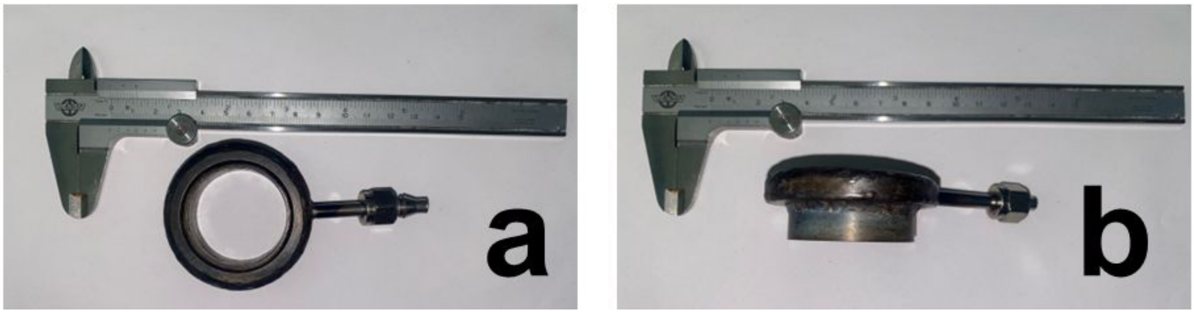

Figure S3. Top (a) and side (b) view of torus ring used here for injection of O_2-containing gas along with a ruler for reference.

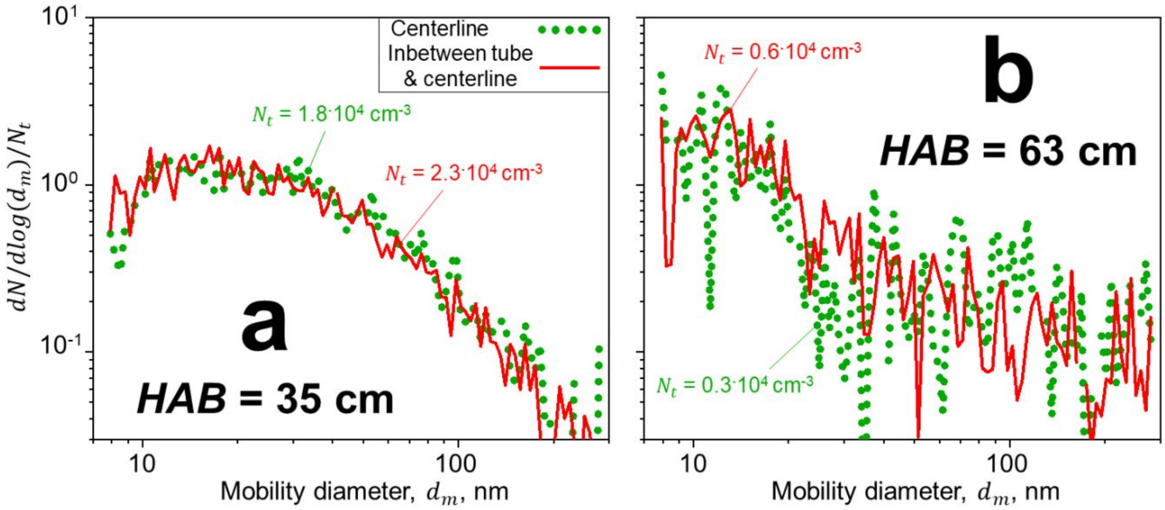

Figure S4. Normalized mobility size distributions of soot from ESC of jet A1 fuel and oxidized by flowing through a torus ring 12 upwards-angled swirling N_2 jets having $[O_2]$ = 20 vol % at the flame centerline (r/R = 0, dotted lines) and in-between enclosing tube wall & centerline (1 cm away from each, r/R = 0.5; solid lines) at *HAB* of 35 (a) and 63 cm (b) along with the corresponding total particle concentrations, N_t.

Table S1. Soot N_t, f_v and $\bar{d}_m$ at the centerline (r/R = 0) and in-between there and tube wall (r/R = 0.5) at *HAB* = 25 cm which is 5 cm below the torus ring.

		Centerline, $r/R = 0$		In-between centerline and tube wall, r/R=0.5
N_t, $\cdot 10^6$ cm^{-3}		3.4 (2.4 - 5.1)		7.1 (4.9 - 10.2)
f_v, $\cdot 10^{-10}$		3.5 (2.4 -5.1)		9.3 (6.9 - 12.6)
$\bar{d}_m$, nm		100.5 (98.6 - 102.6)		113.2 (109.7 - 116.9)

Table S2. Soot N_t and $\bar{d}_m$ at the centerline (r/R = 0) and in-between there and tube wall (r/R = 0.5) at *HAB* = 35 and 63 cm.

		HAB = 35 cm			63 cm	
		Centerline, $r/R = 0$	In-between centerline and tube wall, r/R=0.5		Centerline, $r/R = 0$	In-between centerline and tube wall, r/R=0.5
N_t, $\cdot 10^4$ cm^{-3}		1.8 (0.2 - 6.6)	2.3 (0.6 - 4.8)		0.3 (0.03 - 2.9)	0.6 (0.1 - 4.6)
$\bar{d}_m$, nm		33.4 (27.4 - 54.6)	31 (24.6 - 38.4)		31.5 (8.9 - 73.8)	27.2 (15.6 - 52.8)

References:

S1 Commodo, M., Serra, G., Bocchicchio, S., Minutolo, P., Tommasini, M. & D'Anna, A. Monitoring flame soot maturity by variable temperature Raman spectroscopy. *Fuel* **321**, 124006 (2022) https://doi.org:10.1016/j.fuel.2022.124006.
